# The COMT Val158Met Polymorphism and Reaction to a Transgression: Findings of Genetic Associations in Both Chinese and German Samples

**DOI:** 10.3389/fnbeh.2018.00148

**Published:** 2018-08-03

**Authors:** Cornelia Sindermann, Ruixue Luo, Yingying Zhang, Keith M. Kendrick, Benjamin Becker, Christian Montag

**Affiliations:** ^1^Department of Molecular Psychology, Institute of Psychology and Education, Ulm University, Ulm, Germany; ^2^MOE Key Laboratory for Neuroinformation, The Clinical Hospital of Chengdu Brain Science Institute, University of Electronic Science and Technology of China, Chengdu, China

**Keywords:** vengefulness, Avoidance Motivation, COMT Val158Met, China, Germany

## Abstract

After a transgression, people often either tend to avoid the transgressor or seek revenge. These tendencies can be investigated via a trait approach and surprisingly little is known about their biological underpinnings. One promising candidate gene polymorphism, which may influence individual differences in avoidance of a transgressor and vengefulness, is the COMT Val158Met (rs4680) polymorphism known to affect dopaminergic signaling and among others brain activity in situations in which people punish others for their behavior. We therefore investigated the molecular genetics of individual differences in Avoidance Motivation and vengefulness with a focus on this polymorphism. Possible genetic associations were first investigated in a sample of *N* = 730 Chinese participants (*n* = 196 females) using buccal cells to extract the DNA for genotyping. To replicate the findings we carried out a parallelized investigation in a sample of *N* = 585 German participants (*n* = 399 females). Chinese and German versions of the TRIM-12 and the Vengeance Scale were implemented to assess individual differences in tendencies to react to a transgression. Results show that Met allele carriers of the COMT Val158Met polymorphism (Val/Met and Met/Met) score significantly higher on the tendency to avoid a transgressor in the Chinese male and female samples, with an especially pronounced effect in the female subgroup. The same effect could be found in the German sample, again especially in females. Additionally, carrying a Met allele was associated with higher vengefulness in the Chinese sample only, especially in males. The present findings indicate that the COMT Val158Met polymorphism might influence individual differences in the motivation to avoid transgressors across cultures, especially in females. However, its association with vengefulness seems to be more complex and may exhibit some cultural and gender specific effects.

## Introduction

There are many different potential reactions to a transgression, although the main ones are for the victim to forgive the transgressor, avoid him/her or take revenge. Acts of revenge taking are reported across the globe and also vengefulness as a personality trait has already been investigated in different cultures (e.g., Henrich et al., 2006; Sindermann et al., 2018). Quantitative genetic studies have revealed that individual differences in reactive aggression (heritability estimate: 39%), the reaction to unfair offers (punishing an opponent for an unfair offer; additive genetic effects explained 42% of the variance) and antisocial personality traits such as various types of aggression (heritability estimates: 26–56%), possess a significant heritable component (Vernon et al., 1999; Brendgen et al., 2006; Wallace et al., 2007). This leads to the conclusion that also variance in trait vengefulness might be explained partly by genetic underpinnings. Currently however, surprisingly little is known about the genes/genetic polymorphisms influencing individual differences in trait vengefulness.

To search for possible candidate gene polymorphisms in the context of trait vengefulness, one can take a closer look at revenge behavior. In comparison with trait vengefulness, there are several studies investigating the neurobiological and genetic basis of revenge taking and associated behaviors (e.g., altruistic punishment) in laboratory settings (e.g., Sanfey et al., 2003; De Quervain et al., 2004; Strobel et al., 2011). In these studies, the dopaminergic reward system appears to be of particular importance since punishing others for unfair behavior (which can be seen as a kind of revenge taking) was associated with activation in brain areas such as for example the (dorsal) striatum, nucleus caudatus (NC), and the (anterior) insula (Sanfey et al., 2003; De Quervain et al., 2004). These brain areas have also been associated with reward processing or its anticipation (e.g., Delgado et al., 2003; Liu et al., 2011; Sescousse et al., 2013) and their activity is known to be modulated by dopaminergic pathways (Gaspar et al., 1989; Arias-Carrión et al., 2010). This suggests that pathways and thereon genes and genetic polymorphisms influencing the dopaminergic reward system are associated with acts of revenge and may therefore also be associated with trait vengefulness.

In this regard, one study in the field is of particular interest. Strobel et al. (2011) observed that brain regions involved in reward processing (cingulate gyrus (CG), insula, dorsolateral prefrontal cortex (DLPFC), nucleus accumbens (NAc), NC) were more strongly activated during trials in which participants punished a person who made an unfair offer compared to trials in which participants did not punish anybody. Additionally, the activation of the NAc and bilateral CG was also higher in trials in which participants punished a person who initially made the unfair offer to the participants themselves (revenge), compared to trials in which participants punished a person, which made an unfair offer to a third person (altruistic punishment). Notably, this study also found that the genotype of the Catechol-O-Methyltransferase (COMT) Val158Met (rs4680) polymorphism modulated the neural responses. In detail, it was found that the activation in reward related brain areas (clusters of the left anterior cingulate cortex (ACC), right posterior insula, right NAc) in the punishment trials (vs. no punishment) was higher in Met allele carriers compared to (homozygote) Val allele carriers. This polymorphism is positioned in the COMT gene on chromosome 22q11 (https://www.ncbi.nlm.nih.gov/projects/SNP/snp_ref.cgi?rs=4680) and causes a valine to methionine (G → A) substitution. Moreover, this polymorphism is functional on a biological level as it moderates the activity of the COMT enzyme and thereby dopamine catabolization in the synaptic cleft. This mechanism is of special importance in the prefrontal cortex (PFC) due to a paucity of dopamine transporters (Lewis et al., 2001; for overviews see Mier et al., 2010; Montag et al., 2012). More specifically, it was found that the enzyme activity of Val/Val (GG) carriers was around 38% higher than of Met/Met (AA) carriers in the DLPFC (Chen et al., 2004). Moreover, it was demonstrated that Val/Val (GG) carriers catabolize three to four times more dopamine than Met/Met (AA) carriers. Heterozygote Val/Met (GA) carriers show an intermediate catabolization rate (Lachman et al., 1996). On a psychological level, the COMT Val158Met polymorphism has also been associated with various phenotypes. For example the Met allele of the COMT Val158Met polymorphism is linked to higher reward responsiveness and reward seeking (Lancaster et al., 2012), and higher activity in reward related brain areas (ventral Striatum, lateral PFC) during anticipation of uncertain reward (Dreher et al., 2009). Moreover, it is important to highlight that the different alleles of the COMT Val158Met polymorphism have been associated with individual differences in cognitive abilities and (personality) traits related to stress processing / negative emotionality. In detail, the Met allele seems to be associated with higher abilities in executive cognitive abilities and lower activations in prefrontal brain regions compared to the Val allele while performing cognitive tasks (Mier et al., 2010). In addition, the Met allele of the COMT Val158Met polymorphism has been associated with emotional instability and lower stress resiliency according to the warrior-worrier-hypothesis (but therefore better cognitive functions; Goldman et al., 2005; Montag et al., 2012) and with anxiety and related traits in empirical studies (e.g., Olsson et al., 2005; Stein et al., 2005; Hashimoto et al., 2007; Montag et al., 2008; Lee and Prescott, 2014).

Overall, the results indicate that the COMT Val158Met polymorphism genotype modulates reward processing in association with punishment of unfair behavior and taking revenge. Accordingly, it can be hypothesized that this effect is transferable from revenge-like behavior to trait vengefulness and that Met allele carriers might experience revenge as more rewarding. We therefore hypothesized that carrying the Met allele would be associated with higher scores in trait vengefulness. Since avoiding negative outcomes is also associated with activation in reward related brain areas, we additionally hypothesized that carrying the Met allele would be associated with the tendency to avoid a transgressor (please note that Revenge Motivation and Avoidance Motivation show strong positive correlations and can therefore not be seen as opposites; Kim et al., 2006; Johnson et al., 2010; Szcześniak and Soares, 2011). With the present study we aimed at investigating these hypotheses.

## Materials and methods

### Sample

For the present genetic association studies, data collection was conducted in China and in Germany to independently replicate findings (see also for example Montag et al., 2017). Taking into account (i) power analyses but also (ii) sample sizes of other recent genetic association studies using a questionnaire approach to assess traits and (iii) the independent replication as implemented in the present research led to the conclusion that around 500–600 participants per sample (China and Germany) should be investigated. The complete procedure including the software to present the questionnaires as well as the equipment and protocols for genotyping were the same in China and Germany. Participants in China and Germany were recruited at universities. All Chinese participants are part of the Chengdu Gene Brain Behavior Project and all German participants of the Ulm Gene Brain Behavior Project. For a total of *N* = 730 Chinese participants [*n* = 534 males, *n* = 196 females; mean age: 21.62 (*SD* = 2.35)] complete data were available for the present study. Most of the Chinese participants were Han Chinese (*n* = 680). Additionally, the data of *N* = 585 German participants, of which all stated German as their native language, were included in the analyses of the German part of the study [*n* = 186 males, *n* = 399 females; mean age: 23.74 (*SD* = 8.33)]. All participants gave electronic and written informed consent in accordance with the Declaration of Helsinki. The studies/protocols were approved by the local ethics committees at University of Electronic Science and Technology of China, Chengdu, China and Ulm University, Ulm, Germany.

### Self-report measures

To measure individual differences in the tendencies to react to a transgression two different self-report measures were assessed via an online platform. Participants completed a Chinese/German version of the Transgression Related Interpersonal Motivations−12 (TRIM-12) inventory (original English version: McCullough et al., 1998; McCullough and van Oyen Witvliet, 2002). This measure consists of 12 items split into two scales named Revenge Motivation [5 items; Cronbach's α = 0.87/0.87 (Chinese sample/German sample)] and Avoidance Motivation [7 items; α = 0.83/0.88 (Chinese sample/German sample)]. Answers are given on a 5-point Likert scale. Participants also completed a Chinese/German version of the Vengeance Scale (original English version: Stuckless and Goranson, 1992). It consists of 20 items forming a single scale and the items are answered on a 7-point Likert scale [α = 0.90/0.93 (Chinese sample/German sample)]. For all scales, higher scores indicate higher tendencies toward vengefulness/avoidance of transgressors. The questionnaires used were the same as in Sindermann et al. (2018), where also full details about the Chinese and German translations of the items, the factorial structure of all scales in the Chinese and German languages as well as associations with prominent personality factors are given. It should be noted that some of the participants from the Sindermann et al. (2018) study are also included in the present study (Chinese sample: *n* = 234; German sample *n* = 182).

### Genotyping

DNA was extracted from buccal cells on a MagNA Pure 96 robot (Roche Diagnostics, Mannheim, Germany) using commercial extraction kits. Genotyping of the COMT Val158Met Single Nucleotide Polymorphism (SNP) was conducted on a Cobas Z 480 Light Cycler (Roche Diagnostics, Mannheim, Germany) by means of polymerase chain reaction and subsequent high resolution melting. Simple probe assay designs by TIBMolBiol (Berlin, Germany) were used. Hardy Weinberg Equilibriums (HWEs) were calculated using the Court lab–HW calculator.

### Statistical analyses

The distributions of all scales under investigation were tested for normal distribution (separately in the Chinese and German sample). The statistical tests as well as histograms are presented in the Supplementary Material. As none of the scales was normally distributed according to statistical tests and because especially the histograms of the German sample showed a marked deviation from the normal distribution, it was decided to implement all statistical tests using non-parametric tests.

First, associations of age and gender with all scales were investigated to examine possible confounding variables. Spearman correlations between age and Revenge Motivation, Avoidance Motivation and the Vengeance Scale were calculated and Mann-Whitney *U*-Tests were used to examine gender differences.

To investigate the relationship between the COMT Val158Met polymorphism and the scales under investigation, groups of Met– (A–: GG) and Met+ (A+: GA/AA) carriers were built. This procedure is justified because in the Chinese sample the distribution of genotypes in the COMT Val158Met polymorphism was skewed with only a few individuals (*n* = 50) carrying the homozygote Met/Met (AA) genotype (see Table 1). This is also in line with the expected distribution of the COMT Val158Met polymorphism in the Han Chinese population as indicated by data banks (for further information see also the results section). Therefore, in order to enhance statistical power it is reasonable to group Val/Met (GA) and Met/Met (AA) genotypes into one group called Met+. Despite a similar number of homozygote Val/Val and homozygote Met/Met carriers in the German sample, we implemented the same grouping (Met– vs. Met+) for the data to allow a direct comparison between the Chinese and German samples. Next, we calculated Mann-Whitney *U*-Tests in the Chinese and German samples to investigate differences between the Met– (A–: GG) and Met+ (A+: GA/AA) groups. Additionally, we calculated the Mann-Whitney *U*-Tests separately within the samples from each nation and split by gender (this is necessary due to (i) significant effects of gender on the investigated scales and (ii) with regard to the different gender ratios in the Chinese and German samples). Moreover, we also present all the results (using Kruskal-Wallis-Tests) on a genotype level with the grouping Val/Val (GG), Val/Met (GA), Met/Met (AA) in the Supplementary Material.

**Table 1:** Genotype distributions of the COMT Val158Met polymorphism in the Chinese and German samples.

	**Val/Val (GG)**	**Val/Met (GA)**	**Met/Met (AA)**	**HWE**
China	385	295	50	*X*^2^ = 0.41, *p* = 0.520
Germany	127	314	144	*X*^2^ = 3.24, *p* = 0.072

*HWE = Hardy Weinberg Equilibrium*.

## Results

### Genotype distribution and hardy weinberg equilibriums

As seen in Table 1, the distributions of the genotypes in the COMT Val158Met polymorphism were in the HWE in both samples from China and Germany. The distribution of genotypes also fits with the observations reported at dbSNP in the samples from both nations (https://www.ncbi.nlm.nih.gov/projects/SNP/snp_ref.cgi?rs=4680).

### Associations with age and gender

Only in the Chinese sample age correlated weakly significantly with Avoidance Motivation (ρ = 0.09, *p* = 0.011), but no other scale. In the German sample, age did not significantly correlate with any of the scales of interest (all *p*-values > 0.281). Therefore, it was decided to not include this variable as control variable in further analyses.

Gender differences were found in Avoidance Motivation (*U* = 43,195.50, *Z* = −3.63, *p* < 0.001) in the Chinese sample and in Revenge Motivation (*U* = 28,436.00, *Z* = −4.57, *p* < 0.001) and the Vengeance Scale (*U* = 28,692.00, *Z* = −4.42, *p* < 0.001) in the German sample. Descriptive statistics are presented in Table 2.

**Table 2:** Descriptive statistics of all scales under investigation split by nation and gender.

	**Total**	**Males**	**Females**
**CHINA**			
Revenge Motivation	15.44 (4.19)	15.56 (4.16)	15.11 (4.26)
Avoidance Motivation	25.95 (4.57)	25.58 (4.56)	26.97 (4.48)
Vengeance Scale	69.72 (16.69)	69.89 (16.57)	69.28 (17.03)
**GERMANY**			
Revenge Motivation	10.86 (4.38)	11.89 (4.10)	10.38 (4.43)
Avoidance Motivation	25.03 (5.93)	25.01 (5.81)	25.04 (5.99)
Vengeance Scale	57.27 (17.51)	62.16 (18.24)	55.00 (16.71)

*Mean values and SDs are reported [M (SD)]*.

### Effects of the COMT Val158Met polymorphism

As presented in Table 3, significant effects of the COMT Val158Met polymorphism (tested on Met– vs. Met+ allele level) on Revenge Motivation and the Vengeance Scale were observed, but only in the Chinese sample. Met allele carriers (Met+; A+; GA/AA) showed higher scores than the Met– (Val/Val; A–; GG) group. The strongest significant effect of the COMT Val158Met polymorphism was found on Avoidance Motivation in the Chinese sample with Met allele carriers (Met+; A+; GA/AA) showing higher scores than the Met– group (Val/Val; A–; GG). Results with regard to the effect on Avoidance Motivation were significant and in the same direction in the German sample. In the Chinese sample, all effects of the COMT Val158Met polymorphism would survive Bonferroni correction for multiple testing (α = 0.05/3 = 0.0167; accounting for the number of scales under investigation). In the German sample, the association between the COMT Val158Met polymorphism and Avoidance Motivation would also survive Bonferroni correction for multiple testing (α = 0.05/3 = 0.0167; accounting for the number of scales under investigation).

**Table 3:** Effects of the COMT Val158Met polymorphism (on allele level) on all scales under investigation split by nation.

	**Met– (A–)**	**Met+ (A+)**	**Effect (*U*)**	**Effect (*Z*)**	**Significance**
**CHINA**					
Revenge Motivation	15.08 (4.15)	15.83 (4.20)	*U* = 59,039.00	*Z =* −2.60	*p* = 0.009
Avoidance Motivation	25.46 (4.51)	26.50 (4.58)	*U* = 55,963.00	*Z* = −3.69	*p* < 0.001
Vengeance Scale	68.06 (16.19)	71.58 (17.06)	*U* = 59,058.50	*Z =* −2.59	*p* = 0.010
**GERMANY**					
Revenge Motivation	10.87 (4.49)	10.86 (4.35)	*U* = 28,905.50	*Z =* −0.11	*p* = 0.916
Avoidance Motivation	23.75 (6.09)	25.38 (5.84)	*U* = 24,755.50	*Z* = −2.57	*p* = 0.010
Vengeance Scale	57.21 (16.93)	57.29 (17.69)	*U* = 28,908.00	*Z =* −0.10	*p* = 0.917

*Mean values and SDs are reported [M (SD)] in the first columns. All p-values presented in this table are derived from two-sided testing*.

**Figure 1:**
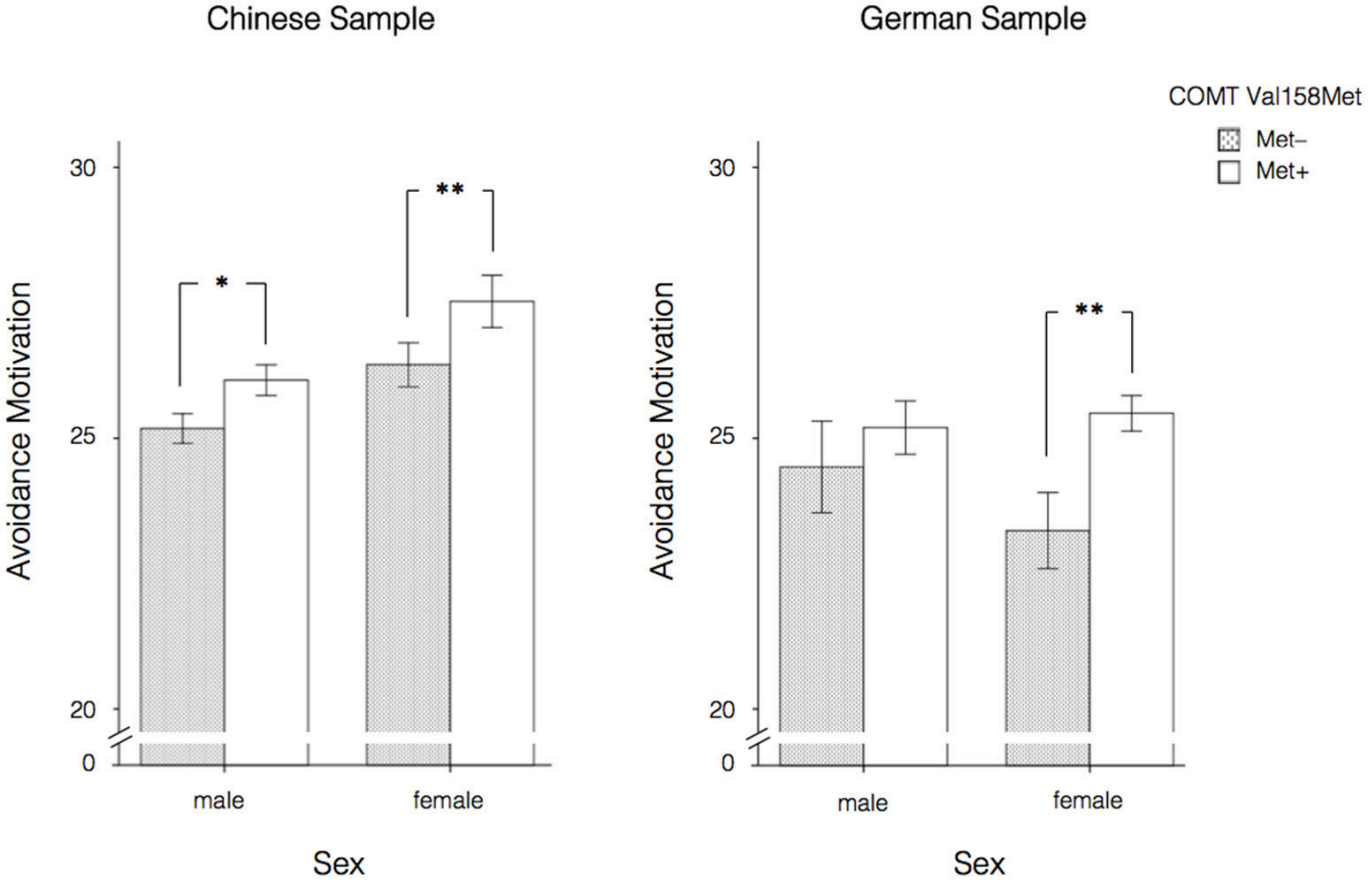
Effects of the COMT Val158Met polymorphism (allele level) on Avoidance Motivation split by nation and gender. Error bars indicate ±1 SE. *p < 0.05, **p < 0.01, two-sided.

When effects were considered separately for the samples from both nations and split by gender, Table 4 shows that the direction of the effects was the same for males and females in the Chinese sample, although the associations with Revenge Motivation and the Vengeance Scale were only significant in males. For all the scales, Met allele carriers (Met+; A+; GA/AA) showed higher scores compared to the Met– group (Val/Val; A–; GG). There were no significant effects of the COMT Val158Met polymorphism on any of the scales under investigation in the German male sample (also when testing one-sided based on the hypotheses and the results in the Chinese sample). However, in the German female sample the effect on Avoidance Motivation was significant and in the same direction as in the Chinese male and female samples. Of note, only the associations between the COMT Val158Met polymorphism and Revenge Motivation and Avoidance Motivation found in the Chinese male sample and the association between the COMT Val158Met polymorphism and Avoidance Motivation in Chinese and German females would still be significant after Bonferroni correction for multiple testing (α = 0.05/3 = 0.0167; accounting for the number of scales under investigation). Additionally, when correcting for multiple testing while accounting for the number of scales under investigation as well as gender, only the association between the COMT Val158Met polymorphism and Revenge Motivation found in the Chinese male sample and between the COMT Val158Met polymorphism and Avoidance Motivation found in the German females would still be significant (α = 0.05/6 = 0.0083). The association between the COMT Val158Met polymorphism and Avoidance Motivation in the Chinese female sample just failed to remain significant (with a *p*-value of 0.009). The strongest effects, namely the ones on Avoidance Motivation are also presented in Figure 1.

**Table 4:** Effects of the COMT Val158Met polymorphism (on allele level) on all scales under investigation split by nation and gender.

	**Met– (A–)**	**Met+ (A+)**	**Effect (*U*)**	**Effect (*Z*)**	**Significance**
**CHINESE MALES**					
Revenge Motivation	15.13 (4.17)	16.07 (4.09)	*U* = 30,639.00	*Z =* −2.65	*p* = 0.008
Avoidance Motivation	25.18 (4.66)	26.07 (4.39)	*U* = 30,828.50	*Z* = −2.55	*p* = 0.011
Vengeance Scale	68.29 (15.88)	71.81 (17.21)	*U* = 31,425.50	*Z =* −2.20	*p* = 0.028
**CHINESE FEMALES**					
Revenge Motivation	14.92 (4.09)	15.27 (4.41)	*U* = 4,497.50	*Z =* −0.74	*p* = 0.460
Avoidance Motivation	26.35 (3.93)	27.52 (4.87)	*U* = 3,756.00	*Z* = −2.62	*p* = 0.009
Vengeance Scale	67.33 (17.19)	71.03 (16.78)	*U* = 4,202.00	*Z =* −1.48	*p* = 0.138
**GERMAN MALES**					
Revenge Motivation	12.02 (4.38)	11.85 (4.01)	*U* = 3,305.00	*Z =* −0.16	*p* = 0.873
Avoidance Motivation	24.47 (5.92)	25.20 (5.78)	*U* = 3,187.00	*Z =* −0.53	*p* = 0.599
Vengeance Scale	60.45 (18.87)	62.77 (18.03)	*U* = 3,077.50	*Z =* −0.86	*p* = 0.388
**GERMAN FEMALES**					
Revenge Motivation	10.14 (4.44)	10.44 (4.43)	*U* = 11,943.50	*Z =* −0.63	*p* = 0.527
Avoidance Motivation	23.29 (6.19)	25.46 (5.87)	*U* = 9,967.00	*Z* = −2.80	*p* = 0.005
Vengeance Scale	55.18 (15.37)	54.95 (17.04)	*U* = 12,309.00	*Z =* −0.23	*p* = 0.818

*Mean values and SDs are reported [M (SD)] in the first columns. N(Chinese, males, Met−) = 292, n(Chinese, males, Met+) = 242, n(Chinese, females, Met−) = 93, n(Chinese, females, Met+) = 103, n(German, males, Met−) = 49, n(German, males, Met+) = 137, n(German, females, Met−) = 78, n(German, females, Met+) = 321. All p-values presented in this table are derived from two-sided testing*.

As a final note, based on the hypotheses derived in the introduction part of the manuscript, also one-sided testing of the COMT Val158Met effects on all scales would be justified and would lead to halved *p*-values. Thereon, also the association between the COMT Val158Met polymorphism and Avoidance Motivation in Chinese males and females would survive the strict Bonferroni correction procedure for multiple testing while accounting for the number of scales under investigation and gender (α = 0.05/6 = 0.0083).

## Discussion

Investigating the genetic underpinnings of individual differences in the tendencies to react to a transgression in independent Chinese and German samples, we found that the COMT Val158Met polymorphism is associated with Avoidance Motivation in several (sub)samples. In the Chinese and the German sample Met allele carriers (Met+; A+; GA/AA) reported higher scores in the tendency to avoid a transgressor as compared to homozygote Val/Val (Met–; A–; GG) carriers. In the Chinese sample, this effect was found in both males and females, but stronger in females. In the German sample, the direction of the association was also observed in males and females, although it was stronger (and only significant) in females again; whereas in males only a small descriptive difference in the same direction was found when comparing Met+ and Met– carriers. Of note, when correcting the alpha level for the number of scales under investigation and gender (α = 0.05/6 = 0.0083), the associations between the COMT Val158Met polymorphism and Avoidance Motivation found in Chinese males and females would only survive if testing one-sided based on the hypothesis formulated in the introduction part of the manuscript.

In the Chinese male sample, we also found an association between the COMT Val158Met polymorphism and vengefulness (Revenge Motivation as well as the Vengeance Scale). Again, Met allele carriers (Met+; A+; GA/AA) showed higher vengefulness scores. In the German sample, on the other hand, we could not find such an association (even if testing one-sided based on the hypothesis). Thus, as we hypothesized, both the tendency to avoid transgressors and to react vengefully toward them are associated with the COMT Val158Met polymorphism, although it should be emphasized that the latter finding was only significant in the Chinese (male) sample.

In sum, the association between the Met allele of the COMT Val158Met polymorphism and the scale Avoidance Motivation was the most stable and robust finding, especially across the two female samples with completely different cultural backgrounds. This indicates a general cross-cultural effect, and maybe gender-specificity. We also want to mention that an independent replication of associations, such as the one reported here, especially in ethnically and cultural different samples, is difficult to observe. Thus, in the present study the independent replication of the effect, mainly in the female subsamples, increases confidence in the overall robustness of our finding. Hence, we argue for the importance of these independently derived effects in the same direction, but also mention that results in the German male sample were particularly weak and not significant. Effects of single genetic markers are often weak [around 1% of explained variance in a certain phenotype (often lower)] and therefore the non-significant findings in the German male sample could be due to a lack of power (with *n* = 186, this sample was the smallest subsample in this study; in detail, in the German male sample *n* = 49 participants were in the Met– group and *n* = 137 in the Met+ group, whereas for example in the Chinese female sample (*n* = 196), we tested *n* = 93 (Met–) vs. *n* = 103 (Met+) participants). However, in line with our findings the effect of the COMT Val158Met polymorphism on Avoidance Motivation might be particularly strong in females. Support for this interpretation comes from previous studies demonstrating gender specific effects of the COMT Val158Met polymorphism on various traits, cognitive abilities and cortical thickness (e.g., Stein et al., 2005; Lang et al., 2007; Chen et al., 2011; Sannino et al., 2014). To further illuminate this: the COMT enzyme does not only metabolize catecholamines but also methylates catecholestrogens (Harrison and Tunbridge, 2008). Additionally, it has been shown that estradiol can inhibit COMT gene transcription and mRNA expression and thereon also COMT enzyme activity in certain cells (Xie et al., 1999; Jiang et al., 2003). In line with this, studies showed that COMT enzyme activity in the liver and erythrocytes was lower in healthy females compared to healthy males (Fähndrich et al., 1980; Philippu et al., 1981; Boudíková et al., 1990). This indicates potentially higher baseline dopamine levels in females compared to males as COMT catabolizes dopamine [But it needs to be mentioned that this will most likely only apply in COMT active brain regions. Additionally, also this argumentation is limited to the COMT related mechanisms to catabolize dopamine. However, there are several more mechanisms to clear the synaptic cleft from dopamine except the enzymatic mechanism by COMT (e.g. MAO-A,…)]. Hence, in females, which are carrying the Met allele, the reduced COMT enzyme activity by carrying the Met allele together with the (generally) reduced COMT enzyme activity by higher estrogen levels (compared to males) might lead to especially pronounced effects on psychological phenotypes in females compared to males (Lachman et al., 1996; Chen et al., 2004). In detail, the potentially higher dopamine levels in female Met allele carriers especially in COMT active regions of the brain might lead to higher Avoidance Motivation compared to female Val/Val carriers. Support for the influence of estrogens (and perhaps other sex hormones) on the (gender specific) effects of the COMT Val158Met polymorphism also comes from a study, which showed that genetic variation in the COMT gene associated with extreme COMT enzyme reduction [22q11DS patients (only one copy of the COMT gene) carrying the Met allele in the COMTVal158Met polymorphism], was associated with cortical thinning only in females and only after puberty (similar effect also found in genetically modified mice; Sannino et al., 2017). Moreover, another study showed that the Met allele of the COMT Val158Met polymorphism was associated with better performance in a working memory task in males and post-menopausal (but not pre-menopausal) women (Papaleo et al., 2015). These studies strengthen the idea that the hormonal status (with regard to sex hormones) is important for the gender specific effects of the COMT Val158Met polymorphism. However, it needs to be noted that the importance of estrogens in inhibiting COMT enzyme activity with a focus on mechanisms in the brain has been challenged by studies showing that estradiol does not affect (i) COMT activity in the rat brain and (ii) in a glioblastoma cell line (Cohn and Axelrod, 1971; Jiang et al., 2003; see also Harrison and Tunbridge, 2008 for an overview). In conclusion, the exact biochemical underpinnings of the here found results of the COMT Val158Met polymorphism cannot be tested with the present dataset as no further biological marker of interest except the genotype in the COMT Val158Met polymorphism was assessed. In so far this discussion part of our work is speculative.

To assume possible reasons and mechanisms on a psychological level, which underlie the association between the COMT Val158Met polymorphism and Avoidance Motivation, it must be noted that there was a substantial correlation between Avoidance and Revenge Motivation in the present study (China: ρ = 0.36, *p* < 0.001; Germany: ρ = 0.41, *p* < 0.001). Hence, one could conclude that the tendency to avoid a transgressor could reflect the tendency toward seeking to punish transgressors by ending a relationship (e.g., friendship) and thus incorporates a component of vengefulness (e.g., a typical item of Avoidance Motivation is “I cut off the relationship with him/her”; McCullough and van Oyen Witvliet, 2002). In this case, the association between the Met allele of the COMT Val158Met polymorphism and higher scores in Avoidance Motivation might be explained by higher experience of reward during punishment of the transgressor (see experimental studies e.g., Sanfey et al., 2003; De Quervain et al., 2004; Strobel et al., 2011; and also the following studies: Gaspar et al., 1989; Delgado et al., 2003; Arias-Carrión et al., 2010; Liu et al., 2011; Sescousse et al., 2013). On the other hand, at least in the German sample, the association of the COMT Val158Met polymorphism with Avoidance Motivation differed from that with vengefulness. This could indicate different underlying mechanisms for the associations of the COMT Val158Met polymorphism with vengefulness compared with its association with Avoidance Motivation. As such, avoiding an aversive stimulus/outcome can also be understood as rewarding by itself (Kim et al., 2006) and higher reward responsiveness has previously been associated with the Met allele of the COMT Val158Met polymorphism (Dreher et al., 2009; Lancaster et al., 2012). Hence, also in this way one could explain the association found in the present study. Next, in accordance with the warrior-worrier hypotheses mentioned above (Goldman et al., 2005) and as the Met allele has also been associated with anxiety and related traits previously (e.g., Olsson et al., 2005; Stein et al., 2005; Hashimoto et al., 2007; Lee and Prescott, 2014), we shortly wanted to test post-hoc whether neuroticism (as an indicator of emotional instability) would mediate the effects of the Met allele of the COMT Val158Met polymorphism on Avoidance Motivation. More detailed information and results are reported in the Supplementary Material. In short, at least neuroticism as measured with the NEO-FFI (Costa and McCrae, 1992) does not seem to explain the present findings. But in this regard it is still important to note that for several anxiety related traits, gender specific effects of the COMT Val158Met polymorphism with especially pronounced results in females (Met allele associated with higher anxiety related traits) have been observed (e.g., Olsson et al., 2005; Stein et al., 2005). Hence, the hypothesis that the association between the Met allele of the COMT Val158Met polymorphism is associated with Avoidance Motivation via an anxiety related trait seems likely in the light of the present gender specific effects already discussed above. In conclusion, there are different ways to explain the present results. Thereon, future large-scale studies investigating the relationship between the COMT Val158Met polymorphism and Avoidance Motivation are needed. In this regard it would be of great interest to investigate the exact motivation/motives for why participants tend to avoid transgressors and how this might relate to reward responsiveness and/or specific anxiety-related traits.

In terms of the relationship between the Met allele of the COMT Val158Met polymorphism and vengefulness, which could only be observed in the Chinese (male) sample, it would be of great interest to investigate possible confounding variables contributing to this apparent cultural (and gender) specificity. Next to catecholestrogens, which might explain gender differences, for example, also cross-cultural differences in societal norms and manners might contribute to observed effects of the COMT Val158Met polymorphism as well as differences between the Chinese and German samples. As such, the works by Hofsede are of potential importance, revealing differences in power distance and individualism/collectivism between both countries, with China scoring higher on collectivism and power distance (https://www.hofstede-insights.com/country/china/; https://www.hofstede-insights.com/country-comparison/germany/). In this regard, it may be important to consider both normative and evaluative assessments of tendencies toward these constructs (as outlined by Montag et al., 2016; Sindermann et al., 2018). Next, other objective measures might be additionally used in future studies to expand the knowledge gained by the present results. As such, it could be considered to assess tendencies to react to a transgression on the behavioral and neural level to overcome some shortcomings associated with self-report measures. Lastly, another very interesting research question would be if / to what extent other dispositions to react to a transgression, for example forgivingness, are influenced by genetic markers such as the COMT Val158Met polymorphism.

In conclusion, the present results show for the first time that there seems to be an association between the Met allele of the COMT Val158Met polymorphism and the personality trait of the tendency to avoid a transgressor, especially in females. Future research will need to explore the underlying mechanisms (e.g., motives for revenge, anxiety and cultural influences) explaining this association, particularly regarding the importance of specific anxiety-related factors.

## Author contributions

CS and CM planned the design of the present study. CS and RL collected the data from the Chinese part of the study. CS, RL, and YZ conducted the genetic analyses of the Chinese samples. CS supported data collection for the German part of the study and analyzed around half of the genetic samples from the German participants. CS wrote the manuscript and carried out the statistical analyses. YZ checked the statistical analyses. CM worked over the first draft of the manuscript. BB and KMK provided helpful comments and worked over the complete manuscript. All authors approved the final version of the manuscript.

### Conflict of interest statement

The authors declare that the research was conducted in the absence of any commercial or financial relationships that could be construed as a potential conflict of interest.
