# Supplementary material for: The COMT Val158Met Polymorphism and Reaction to a Transgression: Findings of Genetic Associations in Both Chinese and German Samples
Source: Front Behav Neurosci. 2018 Aug 3;12:148. doi: 10.3389/fnbeh.2018.00148 (PMC6088175; doi:10.3389/fnbeh.2018.00148)
Supplement: Supplementary file 1 [file Presentation_1.PDF]

# The COMT Val158Met Polymorphism and Reaction to a Transgression: Findings of Genetic Associations in both Chinese and German Samples

**Cornelia Sindermann, Ruixue Luo, Yingying Zhang, Keith M. Kendrick, Benjamin Becker and Christian Montag**

## Distributions of all Scales under Investigation

Supplementary Table 1:
Statistical tests for normal distribution of all scales under investigation in the Chinese and German samples.

| | Revenge Motivation | | | Avoidance Motivation | | | Vengeance Scale | | |
|---|---|---|---|---|---|---|---|---|---|
| | Statistics | df | *p* | Statistics | df | *p* | Statistics | df | *p* |
| **China** | | | | | | | | | |
| Kolmogorov-Smirnov | 0.07 | 730 | < .001 | 0.11 | 730 | < .001 | 0.07 | 730 | < .001 |
| Shapiro-Wilk | 0.99 | 730 | < .001 | 0.96 | 730 | < .001 | 0.98 | 730 | < .001 |
| **Germany** | | | | | | | | | |
| Kolmogorov-Smirnov | 0.11 | 585 | < .001 | 0.10 | 585 | < .001 | 0.06 | 585 | < .001 |
| Shapiro-Wilk | 0.95 | 585 | < .001 | 0.96 | 585 | < .001 | 0.97 | 585 | < .001 |

Supplementary Table 2:
Skewness and kurtosis of all scales under investigation in the Chinese and German samples.

| | Revenge Motivation | Avoidance Motivation | Vengeance Scale |
|---|---|---|---|
| **China** | | | |
| Skewness | -0.20 (SE=.09) | -0.70 (SE=.09) | 0.60 (SE=.09) |
| Kurtosis | -0.16 (SE=.18) | 1.34 (SE=.18) | 1.29 (SE=.18) |
| **Germany** | | | |
| Skewness | 0.68 (SE=.10) | -0.70 (SE=.10) | 0.60 (SE=.10) |
| Kurtosis | -0.10 (SE=.20) | 0.46 (SE=.20) | 0.76 (SE=.20) |

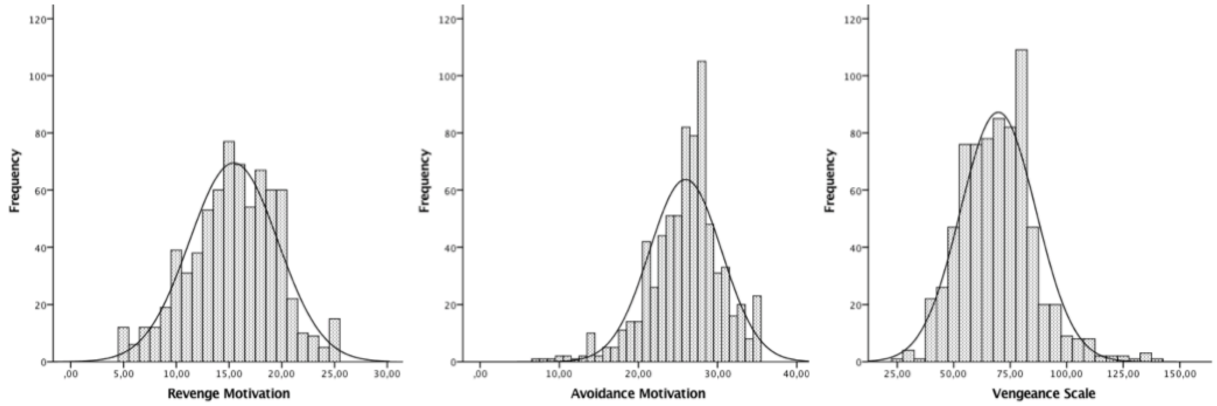

Supplementary Figure 1: Histograms of all scales under investigation in the Chinese sample.

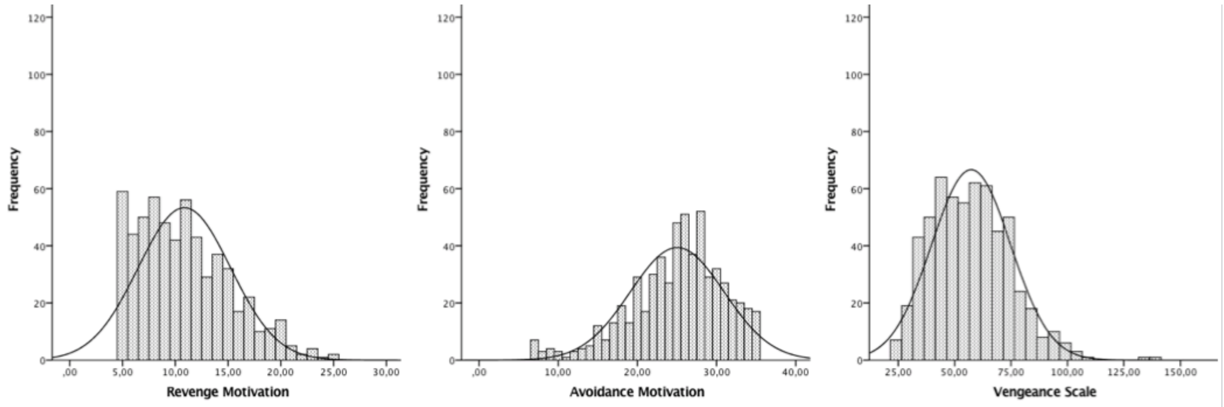

Supplementary Figure 2: Histograms of all scales under investigation in the German sample.

**Effects on Genotype Level**

Supplementary Table 3:
Effects of COMT Val158Met polymorphism (on genotype level) on all scales under investigation split by nation.

| | **Val/Val (GG)** | **Val/Met (GA)** | **Met/Met (AA)** | **Effect** | **Significance** |
|---|---|---|---|---|---|
| **China** | | | | | |
| Revenge Motivation | 15.08 (4.15) | 15.80 (4.34) | 16.04 (3.26) | $X^2(2)=7.06$ | $p$=.029 |
| *Avoidance Motivation* | *25.46 (4.51)* | *26.43 (4.68)* | *26.94 (3.94)* | $X^2(2)=13.78$ | *p=.001* |
| Vengeance Scale | 68.06 (16.19) | 71.25 (17.80) | 73.54 (11.72) | $X^2(2)=8.77$ | $p$=.012 |
| **Germany** | | | | | |
| Revenge Motivation | 10.87 (4.49) | 10.73 (4.36) | 11.14 (4.34) | $X^2(2)=0.89$ | $p$=.640 |
| *Avoidance Motivation* | *23.75 (6.09)* | *25.11 (5.88)* | *25.97 (5.73)* | $X^2(2)=8.78$ | *p=.012* |
| Vengeance Scale | 57.21 (16.93) | 56.61 (17.92) | 58.77 (17.14) | $X^2(2)=2.08$ | $p$=.353 |

Note: Mean values and standard deviations [M (SD)] are reported for Val/Val (GG), Val/Met (GA) and Met/Met (AA) carriers. All *p*-values presented in this table are derived from two-sided testing. The lines written in italics present genetic associations in the same direction in the samples from China and Germany.

Supplementary Table 4:
Effects of COMT Val158Met polymorphism (on genotype level) on all scales under investigation split by nation and gender.

| | Val/Val (GG) | Val/Met (GA) | Met/Met (AA) | Effect | Significance |
|---|---|---|---|---|---|
| **Chinese males** | | | | | |
| Revenge Motivation | 15.13 (4.17) | 15.99 (4.25) | 16.66 (2.85) | $X^2(2)$=8.19 | *p*=.017 |
| *Avoidance Motivation* | *25.18 (4.66)* | *26.03 (4.58)* | *26.34 (2.89)* | $X^2(2)$=6.52 | *p*=.038 |
| Vengeance Scale | 68.29 (15.88) | 71.38 (18.01) | 74.69 (10.29) | $X^2(2)$=7.69 | *p*=.021 |
| **Chinese females** | | | | | |
| Revenge Motivation | 14.92 (4.09) | 15.34 (4.56) | 14.94 (3.73) | $X^2(2)$=0.66 | *p*=.718 |
| *Avoidance Motivation* | *26.35 (3.93)* | *27.42 (4.82)* | *28.00 (5.26)* | $X^2(2)$=6.98 | *p*=.030 |
| Vengeance Scale | 67.33 (17.19) | 70.93 (17.38) | 71.50 (14.00) | $X^2(2)$=2.25 | *p*=.324 |
| **German males** | | | | | |
| Revenge Motivation | 12.02 (4.38) | 12.01 (4.16) | 11.53 (3.71) | $X^2(2)$=0.46 | *p*=.794 |
| Avoidance Motivation | 24.47 (5.92) | 25.13 (6.11) | 25.32 (5.16) | $X^2(2)$=0.29 | *p*=.865 |
| Vengeance Scale | 60.45 (18.87) | 62.58 (19.04) | 63.15 (16.12) | $X^2(2)$=1.01 | *p*=.604 |
| **German females** | | | | | |
| Revenge Motivation | 10.14 (4.44) | 10.22 (4.34) | 10.95 (4.62) | $X^2(2)$=2.22 | *p*=.329 |
| *Avoidance Motivation* | *23.29 (6.19)* | *25.11 (5.80)* | *26.28 (5.98)* | $X^2(2)$=11.32 | *p*=.003 |
| Vengeance Scale | 55.18 (15.37) | 54.21 (16.91) | 56.65 (17.30) | $X^2(2)$=1.34 | *p*=.513 |

Note: Mean values and standard deviations [M (SD)] are reported for Val/Val (GG), Val/Met (GA) and Met/Met (AA) carriers. All *p*-values presented in this table are derived from two-sided testing. The lines written in italics present genetic associations in the same direction in the samples from China and Germany.

**Post-Hoc Analyses about Associations of COMT Val158Met, Vengefulness, and Avoidance Motivation with Neuroticism**

It seems reasonable to consider the tendency towards avoiding a transgressor as an anxiety-related trait, since individuals who are anxious about getting offended again will possibly tend to be more avoidant of previous transgressors. Accordingly and in line with the warrior-worrier hypothesis (Goldman et al., 2005), the Met allele of the COMT Val158Met polymorphism might lead to higher tendencies towards transgressor avoidance due to higher anxiety. This might also provide an alternative explanation why the association between the Met allele and Avoidance Motivation was only found in females in the German sample but in both males and females in the Chinese sample. Findings about the association between the COMT Val158Met polymorphism and anxiety-related traits are inconsistent, and results of various studies (including meta-analyses) suggest that the effects of the COMT Val158Met polymorphism may differ depending on which anxiety-related trait is investigated. Furthermore, these associations appear to be moderated by ethnicity and gender (e. g. Lee & Prescott, 2014; Olsson et al., 2005; Stein et al., 2005). In view of this we carried out a preliminary post-hoc test of this anxiety hypothesis by using the Neuroticism scale of the NEO-FFI, which is known to be associated with anxiety (Costa & McCrae, 1992; Ostendorf & Angleitner, 2004; same translations of the questionnaires as in Sindermann et al., 2016). Data were available for all of the Chinese subjects (N=730), but only for a subsample of n=218 (n=76 males; n=142 females) German subjects. We found that Neuroticism was indeed positively related to Avoidance Motivation, but also to Revenge Motivation and the Vengeance Scale in both Chinese and German males and females (in each sub-sample all the associations were positive, but some only reached significance in the complete sample but not when split by gender). Moreover, we did not find a significant association of the COMT Val158Met polymorphism (neither on genotype nor on allele level) with Neuroticism scores in either Chinese or German males or females. This might reflect the fact that Neuroticism measured by the NEO-FFI does not match anxiety specifically associated with being transgressed repeatedly by the same person. In fact, an anxiety measure assessing anxiety when being transgressed might be of more relevance, here. Moreover, with only 214 participants in the German subsample it is too small to reliably investigate genetic effects, especially when additionally splitting the sample into males and females. This also makes it hard to investigate gene by gender and / or ethnicity effects in the present sample(s).

**References**

Costa, P. T. Jr., and McCrae, R. R. (1992). *Revised NEO Personality Inventory (NEO-PI-R) and NEO Five Factor Inventory (NEO-FFI) Professional Manual*. Odessa, FL: Psychological Assessment Resources.

Goldman, D., Oroszi, G., and Ducci, F. (2005). The genetics of addictions: uncovering the genes. *Nature Reviews Genetics* 6, 521-532. doi: 10.1038/nrg1635

Lee, L. O., and Prescott, C.A. (2014). Association of the Catechol-O-Methyltransferase (COMT) Val158Met Polymorphism and Anxiety-Related Traits: A Meta-Analysis. *Psychiatric genetics* 24, 52-69. doi: 10.1097/YPG.0000000000000018

Olsson, C. A., Anney, R. J., Lotfi-Miri, M., Byrnes, G. B., Williamson, R., and Patton, G. C. (2005). Association between the COMT Val158Met polymorphism and propensity to anxiety in an Australian population-based longitudinal study of adolescent health. *Psychiatric genetics* 15, 109-115.

Ostendorf, F., and Angleitner, A. (2004). *NEO-PI-R - NEO Persönlichkeitsinventar nach Costa und McCrae - Revidierte Fassung* (PSYNDEX Tests Review). Göttingen: Hogrefe.

Sindermann, C., Li, M., Sariyska, R., Lachmann, B., Duke, É., Cooper, A., et al. (2016). The 2D: 4D-Ratio and neuroticism revisited: empirical evidence from Germany and China. *Frontiers in psychology* 7, 811. doi: 10.3389/fpsyg.2016.00811

Stein, M. B., Fallin, M. D., Schork, N. J., and Gelernter, J. (2005). COMT polymorphisms and anxiety-related personality traits. *Neuropsychopharmacology* 30, 2092-2102. doi: 10.1038/sj.npp.1300787
